# Bilateral Simultaneous Pseudoangiomatous Stromal Hyperplasia of the Breasts and Axillae: Imaging Findings with Pathological and Clinical Correlation

**DOI:** 10.1155/2016/9084820

**Published:** 2016-10-27

**Authors:** Afsaneh Alikhassi, Fereshteh Ensani, Ramesh Omranipour, Alireza Abdollahi

**Affiliations:** ^1^Department of Radiology, Cancer Institute, Tehran University of Medical Sciences, Tehran, Iran; ^2^Department of Pathology, Cancer Institute, Tehran University of Medical Sciences, Tehran, Iran; ^3^Division of Surgical Oncology, Cancer Institute, Tehran University of Medical Sciences, Tehran, Iran

## Abstract

Pseudoangiomatous stromal hyperplasia (PASH) of the breast is a pathology that is usually diagnosed by accident during pathological examination of other breast lesions. PASH is an uncommon and benign tumoral lesion of the mammary stroma that can be pathologically mistaken for other tumours, such as phyllodes, fibroadenoma, and sometimes even angiosarcoma. We report the case of a 45-year-old woman with complaints of huge bilateral breast enlargement. This is a rare case of PASH presenting with gigantomastia and involving bilateral breasts and axillae simultaneously. Mammography, ultrasonography, and MRI features are illustrated with histopathological correlation.

## 1. Introduction

First described in 1986 by Vuitch et al. [1], pseudoangiomatous stromal hyperplasia (PASH) of the breast is an uncommon pathology that is usually diagnosed incidentally upon pathological examination of other benign lesions, and it rarely occurs as a growing lump [2, 3]. Huge nodular PASH in the breast and simultaneous axillary tumoral PASH are extremely rare [4, 5]. Only rarely, like in this case, PASH may present with a simultaneous bilateral enlargement of the breast [6, 7]. It is usually found in women, but male cases have been reported [8, 9]. Although its etiology and pathogenesis are still unclear, it is generally thought that PASH represents a neoplastic process of myofibroblasts and the correlations between PASH and hormonal stimuli have been widely discussed in the literature [10].

Upon gross examination, PASH is usually well encapsulated, sometimes lobulated, and usually oval. Histologically, PASH can pose differential diagnostic problems, especially with benign and malignant vascular lesions. As PASH may occasionally adopt a solid fascicular growth pattern, it can be confused with pure mesenchymal spindle cell lesions (myofibroblastoma; leiomyoma; and fibromatosis) or fibroepithelial lesions (fibroadenoma; hamartoma; and phyllodes tumors) containing a spindle cell component [11, 12]. Most patients are premenopausal women, but there are also reports of PASH cases in adolescent girls [13]. PASH in males has also been reported with incidence in as many as 47% of gynecomasty cases [8, 9]. Final diagnosis requires biopsy, and the treatment of choice is excision with wide margins because the rate of local recurrence is quite high [14]. However, it seems to have no association with malignancy, and it does not seem to be a premalignant lesion [15].

## 2. Case Presentation

**Figure 1:**
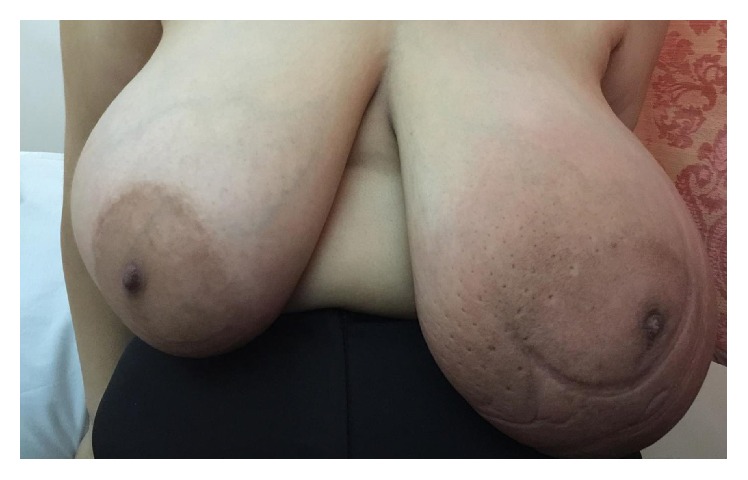
Breasts asymmetric enlargement in a patient with bilateral pseudoangiomatous stromal hyperplasia of the breasts.

A 45-year-old woman was referred to our breast clinic due to slow growing bilateral breast enlargement since 2 years priorly, with more growth on the left, causing significant asymmetry (Figure 1). In clinical examination, both breasts were enlarged with multiple lumps which were soft and mobile upon palpation. The overlying skin was thickened and erythematous on the left side. Soft lumps were also palpable in both axillae. The patient did not have any systemic disease, was premenopausal, had not received any hormonal therapy, and had no family history of breast cancer.

The patient underwent mammography, ultrasound examination, and MRI of the breasts. To obtain samples for pathology, we performed a 14-gauge core needle biopsy of the right breast under ultrasound guidance and excisional biopsy of the left breast. PASH was diagnosed in both methods. Breast tissue with expanded stroma and scattered ducts were seen and immunohistochemical analyses were performed using antibodies against vimentin, CD34, CD31, and alpha-smooth muscle actin. The cells lining the pseudovascular spaces were stained with vimentin and alpha-smooth muscle actin, while they were negative to CD31. These immunohistochemical findings were consistent with the myofibroblastic nature of the cells, supporting the diagnosis of PASH. The patient was informed about the standard treatment of local excision with free margin. The conditions warranted left breast skin-sparing mastectomy, so she refused to undergo surgery, but she wanted to have more time to think about it and is now under observation. Informed consents and permissions were obtained from the patient.

## 3. Discussion

### 3.1. Clinical Presentation

PASH patients can present with mass palpation, abnormality in screen mammography, or incidental pathology finding [16, 17].

### 3.2. Etiology and Risk Factors

The etiological factors of PASH are unknown. A hormone-dependent etiology of PASH has been strongly suggested [11], mostly because of its occurrence in premenopausal women and postmenopausal females receiving hormone replacement therapy, as well as the presence of estrogen and progesterone receptors in most cases of PASH. A well-accepted hypothesis is that the stromal hyperplasia in PASH results from an exaggerated responsiveness of myofibroblasts to hormonal stimuli [1, 10, 12].

### 3.3. Pathology

**Figure 2:**
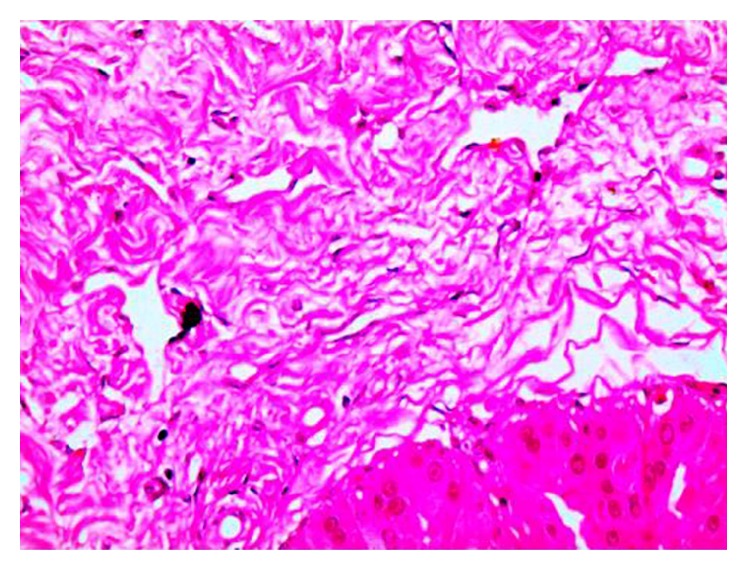
Breast tissue with expanded stroma and scattered ducts in our patient with PASH.

PASH is not a rare pathological diagnosis [15] and can accompany other breast pathologies. However, the tumoral form of PASH is rare. The microscopic appearance of PASH is similar to endothelial spindle cells forming vessel-like slits within the stroma, which are not true vessels covered with endothelia but are vacant spaces bordered by myofibroblasts (Figure 2). The slit-like channels may be mistaken for a low-grade angiosarcoma. However, angiosarcoma can be differentiated based on malignant cytologic features and positive immunohistochemical staining of endothelial markers [14]. Coexisting or subsequent development of carcinoma at the site of PASH and subsequent diagnosis of malignancy in the opposite breast are all possible [1].

### 3.4. Imaging

**Figure 3:**
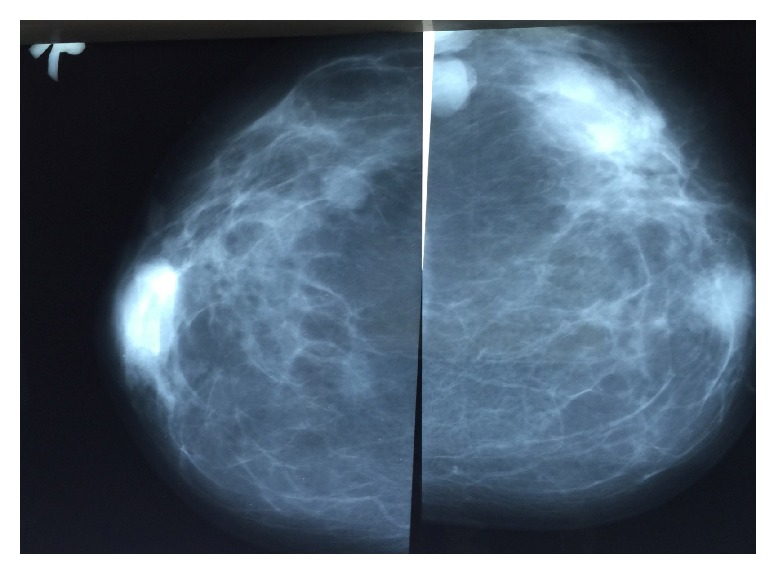
Analogue mammography of the patient two years priorly showing multiple well-defined oval masses or partially defined masses in both breasts.

**Figure 4:**
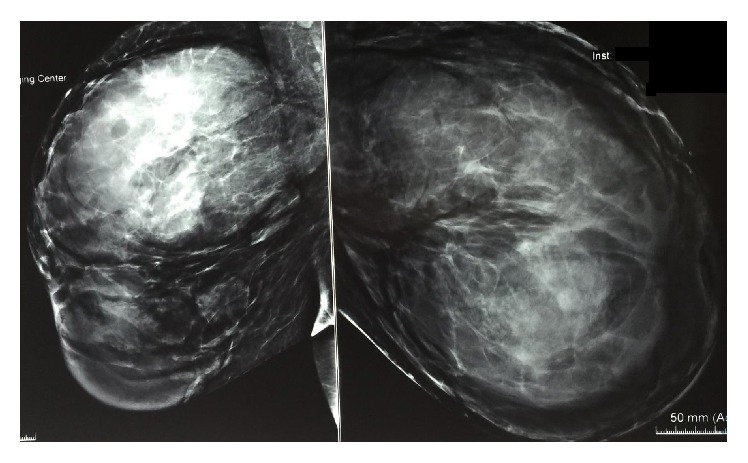
Recent digital mammography of the same patient. Bilateral masses were significantly enlarged compared with previous mammography.

Jones et al. reported mammographically detected abnormality in 78% of cases [17], but another study [18] reported that 69% of patients with PASH presented no mammographic abnormality. However, that series included cases in which PASH was incidentally noted histologically. Mammographic features of PASH can be quite nonspecific and can show a wide range of variation. In mammography, PASH can appear as a partially or well-circumscribed mass or as an asymmetric density. Mass is the most prevalent sign in mammography [13, 16, 19]. In the present case, multiple well-defined and partially ill-defined masses had been seen in both breasts two years priorly, which increased significantly in size in subsequent mammography (Figures 3 and 4).

### 3.5. Sonography

Most patients with dominant PASH pathology have detectable lesions in sonography [17]. Jones et al. reported that the most common sonographic sign is a circumscribed oval hypoechoic mass [17]. PASH findings in sonography are usually nonspecific and are more suggestive of a benign lesion [20–22]. Coexisting fibrocystic changes can result in more heterogeneous appearance [5]. A less commonly reported morphology is a heterogeneous or echogenic area with hypoechoic central areas [21]. Piccoli et al. described this finding in association with a developing focal asymmetry in mammography [21]. Another reported PASH morphology is a mass with irregular or poorly defined borders, which mandates biopsy to rule out malignancy [17].

**Figure 5:**
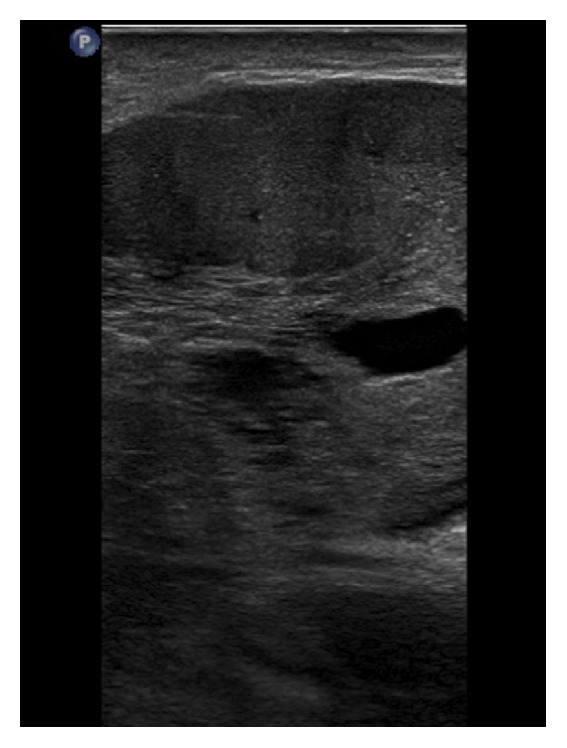
Sonography of the patient's left breast showing hypoechoic mass with solid appearance and multiple cystic changes.

In our patient, multiple similar hypoechoic masses with solid appearance and prominent internal cystic changes were seen bilaterally and in both axillae (Figure 5). The simultaneous bilateral and biaxillary involvement makes this case very notable and rare. It seems that cystic changes inside these solid masses could be an important sonographic sign for nodular PASH.

### 3.6. MRI

**Figure 6:**
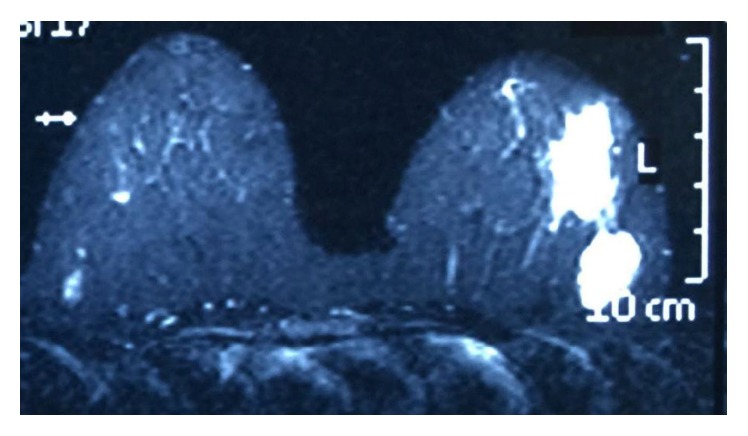
MRI with contrast of the patient shows two intensely enhanced masses with irregular borders in left breast lateral part.

The appearance of PASH has been described in very few cases of MRI [23]. Our patient shows multiple masses with low signal in T1 sequences and high signal in T2 sequences with irregular border or microlobulated border throughout both breasts. This was observed with early homogenous and intense enhancement (Figure 6) with all three types of enhancing curves that are more common for types I (persistent) and II (plateau). Malignant disease could not be excluded in this patient with MRI, which prompted biopsy.

### 3.7. Treatment

Most often, PASH is stable over time [24], but it may increase rapidly in size or recur. High PASH recurrence without complete surgery has been reported [12, 25]. Surgery may warrant a lumpectomy or even a skin-sparing mastectomy. Tumor histology, cosmetic results, and patient preferences should be considered before performing the operation or referring the patient for follow-up at a reference center for breast cancers.

## 4. Conclusion

PASH is a rare benign lesion that can have variable imaging features and may mimic malignant or other benign conditions. Diagnosis requires biopsy. Follow-up or surgery may be warranted, depending on tumoral histology, cosmetic results, risk factors, and patient preference.
